# Practice and Barriers towards Provision of Health Promotion Services among Community Pharmacists in Gondar, Northwest Ethiopia

**DOI:** 10.1155/2017/7873951

**Published:** 2017-07-31

**Authors:** Dessalegn Asmelashe Gelayee, Gashaw Binega Mekonnen, Seyfe Asrade Atnafe

**Affiliations:** ^1^Department of Pharmacology, College of Medicine and Health Sciences, University of Gondar, P.O. Box 196, Gondar, Ethiopia; ^2^Department of Clinical Pharmacy, College of Medicine and Health Sciences, University of Gondar, P.O. Box 196, Gondar, Ethiopia

## Abstract

**Background:**

Health promotion is now becoming an integral part of community pharmacy practice worldwide.

**Objectives:**

This study was intended to determine the level of involvement of community pharmacists in providing health promotion service and to identify the barriers to the practice.

**Methods:**

A cross-sectional study was conducted on 48 community pharmacists working in Gondar town, Northwest Ethiopia. Data on sociodemographic factors, practice, and barriers to health promotion service were collected and analyzed using SPSS version 20.

**Results:**

The majority of respondents were B.Pharm holders (*n* = 27, 56.3%). Almost all respondents (*n* = 45, 95.8%) were willing and claimed to be well involved in health promotion services. The top 5 services were related to drug misuse, asthma, diabetes, family planning, and cardiovascular diseases. However, involvement in some types of health promotion services significantly differed based on sex, educational level, and pharmacy ownership of respondents. The main barrier reported was lack of training.

**Conclusion:**

The community pharmacists in Gondar, Northwest Ethiopia, are engaged in health promotion activities. However lack of training has limited their level of involvement and practice differences were noted based on sex, educational level, and pharmacy ownership status of the respondents.

## 1. Background

Health promotion is now seen as a priority of the health service. According to the World Health Organization (WHO), health promotion refers to a process of enabling people to increase control over, and to improve, their health [1]. Such strategies including discouragement of smoking, alcohol abuse, weight management, and physical activity are necessary to decrease personal and societal consequences as well as improve overall quality of life [2]. Pharmacists can play an important role in promoting the health of individuals. They are the third largest regulated healthcare professional group in the world, and community pharmacy is the most common discipline represented [3]. A 2012 report stated that 55% of pharmacists working in 90 countries were found to work in community pharmacy environments [4]. Being one of the most accessible healthcare practitioners, the community pharmacist can play a major role in the provision of health promotion activities to the society. Community pharmacy as a setting for public health activities has several advantages such as extended opening hours and absence of appointments needed for advice [5].

So far, the pharmacist's role in patient care has been growing as professional and educational standards develop. Therefore health promotion is now becoming an integral part of community pharmacy practice. The Joint International Pharmaceutical Federation (FIP)/WHO guideline on good pharmacy practices incorporates health promotion as one of six components which contribute to the health improvement of individuals that access community pharmacy services [6]. A systemic change has occurred worldwide in community pharmacy practice during the past 20 years. Now, community pharmacists provide many services in addition to drug dispensing: medication therapy management; immunizations for children and adults; screening for diabetes and cardiovascular disease; and health education consultation for a range of health risks and conditions such as diabetes, smoking cessation, weight management, hypertension, osteoporosis, and substance abuse. These practice changes highlight the fact that pharmacies are important partners for the expansion of public health access [7].

Pharmacy practice in Ethiopia has been product oriented for long time and a strategic shift towards patient oriented pharmacy practice took place in 2008 when the undergraduate pharmacy curriculum in public universities was revised into 5-year program of which the final year is for internship [8]. This transition began preparing students for evolving professional roles including health promotion activities. In the nation, noncommunicable diseases (NCDs) accounted for 30% of deaths in 2014 alone. Cardiovascular diseases, cancers, diabetes, and chronic respiratory diseases were responsible for more than 80% of NCD-related deaths and they share four common risk factors: tobacco, insufficient physical activity, unhealthy diet, and excessive alcohol use. Avoiding these risk factors prevent 80% of cardiovascular diseases and diabetes and over a third of cancer related deaths [9]. As experience shows [7], pharmacists can play an active role in reducing the above risk factors. Moreover, most customers of community pharmacies worldwide found the setting convenient for health promotion and felt that pharmacists should provide the services [5].

Despite opportunities and potential benefits of involving pharmacists in public health activities, behavioral changes are needed. Thus pharmacists must accept their role in public health and make the necessary changes in behavior to carry out the service. The general public must also accept pharmacists as providers of public health services and be willing to seek advice on some health issues from pharmacists [5]. To assist these behavioral changes, baseline studies characterizing the involvement of pharmacists and the barriers hindering the health promotion service are very important. There is lack of studies that document this practice and barriers in Ethiopia. Such data are valuable in the development of public health programs and policies that engage community pharmacists. Therefore this study was designed to assess involvement; perceptions on quality and satisfaction; and identity practice barriers and explore associations with demographics of health promotion activities among community pharmacists in Gondar town, Northwest Ethiopia.

## 2. Methods

This cross-sectional study was conducted among community pharmacists working in Gondar town, Northwest Ethiopia, from September to December 2016. The town is located about 727 km northwest of Addis Ababa, the capital city of Ethiopia. According to the 2007 national Central Statistical Agency report, an estimated population of 207,044 live in the town. There were about 53 medication retail outlets (19 pharmacies and 34 drug stores). In this study, community pharmacist refers to at least diploma holders in pharmacy education and community pharmacy refers to both drug stores and pharmacies. The data collection instrument was a structured self-administered questionnaire developed based on literature reviews which is attached as Supplementary Material available online at https://doi.org/10.1155/2017/7873951. It consists of 3 parts: Part 1: eight questions related to sociodemographics which are both open and closed questions; Part 2: involvement in 12 types of health promotion activities with 4 point Likert scale responses (1 = very uninvolved, 2 = uninvolved, 3 = involved, and 4 = very involved); Part 3: six barriers limiting involvement in health promotion services with 4 point Likert scale responses (1 = strongly disagree, 2 = disagree, 3 = agree, and 4 = strongly agree). The questionnaire was assessed and approved for face validity by three senior pharmacists who are academicians and researchers. It was then pretested on 5 pharmacy technicians working as part time in private pharmacies and modifications were made before distributing the questionnaire for data collection. The inclusion criterion was being a community pharmacist for at least one year in the study area and exclusion criterion was unwillingness to be involved in the study. Thus data were collected on 48 respondents, cleared and entered into the computer, and analyzed by using the Statistical Package for Social Sciences (SPSS) version 20.0 for windows (SPSS Inc., Chicago, Illinois). The reliability assessment of the different subcomponents of the questionnaire following data collection revealed a Cronbach's alpha value of 0.800 for the level of involvement in health promotion services (12 items) and 0.695 for the barriers in providing the service (6 items). Data on sociodemographics, level of involvement, and barriers to health promotion activities were analyzed and the results were described in terms of frequencies, percentages, and means. Mann–Whitney *U* test was employed to analyze group differences (based on sociodemographic factors) on the level of involvement in health promotion activities, perception of quality of health promotion service provided, satisfaction with the service provided, and barriers to health promotion activities.

Pearson's Chi-square test of independence was used to assess association between sociodemographic factors and perception that previous professional curricular training is adequate for offering health promotion service. In all tests *P* value < 0.05 is considered statistically significant. An ethical clearance was taken from the Ethical Review Committee of School of Pharmacy, University of Gondar, and all respondents were asked for their consent before participation in the study. All personal information was deidentified prior to data analysis and only aggregates of data were reported.

## 3. Results

Forty-eight (48) pharmacists working in community pharmacies located in Gondar town gave their consent to participate in the study. All completed and returned the questionnaires making a 100% response rate. The majority were male (*n* = 32, 66.7%), 23–28 years old (*n* = 27, 56.3%), at least B.Pharm holders (*n* = 27, 56.3%), with work experience of 4 years and below (*n* = 25, 53.2%), and employee (*n* = 30, 62.5%). Almost all respondents (*n* = 45, 95.8%) were willing to provide health promotion service to the public but 15 (31.3%) respondents opined that the curricular training they received was not sufficient to be involved in health promotion services, Table 1.

When the types of health promotion services the respondents claimed to be involved/very involved were ranked in descending order, counseling services related to drug misuse, asthma, and diabetes were on the top while counseling services related to immunization, traditional medicines, and cancer were the least ones, Table 2.

As shown in Table 3, 21 (43.8%) respondents perceived that the health promotion service they provided was not good/very good and 13 (27.1%) said that they were unsatisfied/very unsatisfied with the service.

The barriers listed in Table 4 were accepted (agree/strongly agree) by 32 (66.7%) to 38 (79.2%) respondents. Lack of training, insufficient management support, and lack of standard guideline were the top three barriers identified in provision of health promotion service.

The majority of respondents from each category of sociodemographic variables unanimously opined that the curricular training was sufficient to provide health promotion services and there was no group difference on this opinion. However significant difference (*P* = 0.03) was found based on pharmacy ownership. Employee pharmacists were 4.7 times more likely to say that curricular teachings they received were sufficient to provide the health promotion services compared to the owners, Table 5.

Group differences were found with regard to the type of health promotion service respondents were involved based on sex, educational level, and pharmacy ownership. Females were significantly more involved than males in providing counseling on nutritional and physical activity (*P* = 0.005), weight management (*P* = 0.010), and cancer (*P* = 0.017). Diploma holders were more involved than degree and above holders in counseling related to immunization (*P* = 0.040) whereas the employees were more involved in weight management counseling service than the owners (*P* = 0.029),. There employee pharmacists were more satisfied than the owners in the type of health promotion service they provided (*P* = 0.017).

There were no group differences based on sociodemographic characteristics on the perceived barriers of health promotion service except for pharmacy ownership. The employee pharmacists were more likely to suggest insufficient managerial support as a barrier than the owners (*P* = 0.019).

## 4. Discussion

Pharmacy practice worldwide has changed and health promotion is now recognized as an integral part of the modern pharmacy practice [10–12] and the same is true for pharmacists in Ethiopia. Almost all the surveyed pharmacists were willing to provide health promotion services to the public. As most of the respondents were young, it is a good opportunity to make interventions and change the traditional practice where pharmacists are less involved in rendering health promotion services.

Except for counseling services on cancer disease and traditional medicine, more than half of the community pharmacists in the present study are involved/very involved in wide range of health promotion services. The top 5 services are related to drug misuse, asthma, diabetes, and family planning as well as cardiovascular diseases. This is an important finding as asthma, diabetes, and cardiovascular diseases are the leading causes of noncommunicable disease related deaths in Ethiopia [9]. However involvement of pharmacists in cancer related health promotion services is relatively minimal and needs to be improved. A similar high involvement of pharmacists in diabetes and asthma related health promotion services were reported among community pharmacists in Malaysia [13]. Offu et al. [14] reported that community pharmacists in Nigeria are also highly involved in screening for diabetes and hypertension. Thus community pharmacists in developing countries are already involved in health promotion services despite lack of information regarding the quality and impact of the service provided. Further studies in these areas are very important and urgently needed to make successful interventions. Nearly half of pharmacists perceived that the quality of health promotion service they provided is poor/fair while less than third reported that they are unsatisfied/very unsatisfied with their services. These findings signify existing gaps on the practice demanding an intervention. It is consistent with previous studies where one-third of surveyed pharmacists in Scotland [10] did not feel being competent as health promoters and in Moldova [15] where the participating pharmacists underscored their competence on health promotion lower than other professional roles. However, majority of pharmacists surveyed in Nigeria felt confident in advising patients on health promotion [12].

The level of involvement of community pharmacists on some types of health promotion services is influenced by their sex, educational level, and pharmacy ownership status. Female pharmacists were significantly more involved than males in providing health promotion services on nutritional and physical activity, weight management, and cancer disease. Diploma holders claimed to be significantly more involved in immunization counseling than better educated pharmacists and the employed pharmacists were significantly more involved than owners in weight management health promotion services. Though the present study assessed self-reported level of involvement of the pharmacists, it seems that male pharmacists were less involved in the practice and thus barriers unique to these groups shall be further studied. Female pharmacists significantly highly rated the quality of service they provided compared to males and the employee pharmacists are significantly more satisfied with their service than the owners.

Nearly 2/3rd of respondents agreed with all the barriers listed in the data collection tool as obstacles to provide the health promotion services. The top three barriers were lack of training, insufficient management support, and absence of standard practice guideline of health promotion for the pharmacists. These findings were consistent with that reported in previous studies [13, 16–18]. Group differences for opinion on barriers for health promotion service were found only for managerial support. Accordingly, employee pharmacists significantly highly rated insufficient management support as a barrier compared to the owners.

The findings of the present study imply that community pharmacists in Ethiopia are involved in health promotion activities despite limitations and uncertainty in its scope and quality.

This study, however, has its own limitation and requires attention in interpreting the findings. Since the method of data collection was self-administered questionnaire, social desirability bias might have happened and inflated the outcomes.

## 5. Conclusion

Counseling services related to drug misuse and asthma were the commonest ones while those of traditional medicine and cancer were the least performed. Quality of service provided was rated as good/very good by the majority of respondents. They were also satisfied/very satisfied with their service. Lack of training was the major barrier identified. The level of involvement of community pharmacists on some types of health promotion services is influenced by sex, educational level, and pharmacy ownership status of the respondents.

## Supplementary Material

This instrument was developed based on previous studies and was uses to collect data from community pharmacists.

## Figures and Tables

**Table 1:** Demographic and additional characteristics of the respondents (*n* = 48).

Variables	*n* (%)
Sex	
Female	16 (33.3)
Male	32 (66.7)
Age (mean = 30.5, median = 28 SD = 6.8)	
23–28 years	27 (56.3)
29–51 years	21 (43.7)
Educational level	
Diploma	21 (43.7)
B.Pharm	26 (54.2)
M.S	1 (2.1)
Work experience in community pharmacy (years) mean = 5.4, median = 4.5, range = 15	
1–4 years	24 (50)
5–16 years	24 (50)
Pharmacy ownership	
Owner	18 (37.5)
Employee	30 (62.5)
Additional work experience	
Yes	23 (47.9)
No	25 (52.1)
Willing to provide health promotion service	
Yes	46 (95.8)
No	2 (4.2)
Think professional curricular training is adequate for offering health promotion service	
Yes	33 (68.7)
No	15 (31.3)

**Table 2:** Involvement of community pharmacists in health promotion activities (*n* = 48).

Involvement in health promotion	Response *n* (%)			
	Very uninvolved	Uninvolved	Involved	Very involved
Drug misuse counseling	1 (2.1)	3 (6.3)	30 (62.5)	14 (29.1)
Asthma counseling	1 (2.1)	5 (10.4)	29 (60.4)	13 (27.1)
Diabetes counseling	0	9 (18.8)	28 (58.3)	11 (22.9)
Family planning counseling	0	11 (22.9)	21 (43.8)	16 (33.3)
Cardiovascular counseling	0	12 (25.0)	24 (50.0)	12 (25.0)
Nutritional and physical activity counseling	2 (4.2)	13 (27.1)	27 (56.3)	6 (12.4)
Oral health counseling	2 (4.2)	15 (31.3)	30 (62.4)	1 (2.1)
Weight management counseling	1 (2.1)	19 (39.6)	24 (50.0)	4 (8.3)
Smoking cessation counseling	2 (4.2)	20 (41.7)	21 (43.7)	5 (10.4)
Immunization counseling	2 (4.2)	22 (45.8)	20 (41.7)	4 (8.3)
Traditional medicine counseling	4 (8.3)	24 (50.0)	18 (37.5)	2 (4.2)
Cancer counseling	3 (6.3)	27 (56.2)	14 (29.2)	4 (8.3)

4-point Likert scale (1 = very uninvolved; 4 = very involved).

**Table 3:** Perception of quality of health promotion service and satisfaction with the service provided (*n* = 48).

Variable	Response *n* (%)			
	Poor	Fair	Good	Very good
Quality of health promotion service provided	7 (14.6)	14 (29.2)	22 (45.8)	5 (10.4)

	very unsatisfied	unsatisfied	satisfied	very satisfied
Satisfaction with the health promotion service provided	4 (8.3)	9 (18.8)	27 (56.3)	8 (16.6)

4-point Likert scale (1 = poor/very unsatisfied; 4 = very good/very satisfied).

**Table 4:** Barriers for provision of health promotion service (*n* = 48).

Barriers to provide health promotion service	Response *n* (%)			
	Strongly disagree	Disagree	Agree	Strongly agree
Lack of training	1 (2.1)	9 (18.8)	29 (60.4)	9 (18.8)
Insufficient management support	1 (2.1)	9 (18.8)	32 (66.6)	6 (12.5)
Absence of standard guideline for the service	0	11 (22.9)	25 (52.1)	12 (25.0)
Lack of time	2 (4.2)	12 (25.0)	28 (58.3)	6 (12.5)
Lack of reimbursement from employer or consumers	3 (6.3)	12 (25.0)	30 (62.4)	3 (6.3)
Lack of profitability	3 (6.3)	13 (27.1)	28 (58.3)	4 (8.3)

4-point Likert scale (1 = strongly disagree; 4 = strongly agree).

**Table 5:** Relationship between demographic factors and opinion whether professional curricular training is adequate for offering health promotion service (*n* = 48).

Variables	Responses	
	Think professional curricular training is adequate for offering health promotion service (*n* = 33)	
	*n* (%)	*P* value (*X*^2^, d.f)
Sex		
Female (*n* = 16)	10 (62.5)	0.509 (0.436, 1)
Male (*n* = 32)	23 (71.9)	
Age (years)		
23–28 (*n* = 27)	18 (66.7)	0.724 (0.125, 1)
29–51 (*n* = 21)	15 (71.4)	
Educational level		
Diploma (*n* = 21)	14 (66.7)	0.784 (0.075, 1)
Degree and above (*n* = 27)	19 (70.4)	
Work experience (years)		
1–4 years (*n* = 24)	18 (75)	0.350 (0.873, 1)
5–16 years (*n* = 24)	15 (62.5)	
Additional work experience		
No (*n* = 25)	18 (72)	0.613 (0.257, 1)
Yes (*n* = 23)	15 (65.2)	
Pharmacy ownership		
Owner (*n* = 18)	9 (50)	0.030^*∗*^ (4.713, 1)
Employee (*n* = 30)	24 (80)	

^*∗*^Statistically significant at *P* < 0.05.

## Abbreviations

NCDs: Noncommunicable diseases
WHO: World Health Organization.
