# Supplementary material for: Practice and Barriers towards Provision of Health Promotion Services among Community Pharmacists in Gondar, Northwest Ethiopia
Source: Biomed Res Int. 2017 Jul 31;2017:7873951. doi: 10.1155/2017/7873951 (PMC5555023; doi:10.1155/2017/7873951)
Supplement: Supplementary file 1 — This instrument was developed based on previous studies and was uses to collect data from community pharmacists. [file 7873951.f1.docx]

**Practice and barriers towards provision of health promotion services among community pharmacists in Northwest Ethiopia: A baseline survey**

**Part I: Socio-demography**

1. Sex: female male
2. Age (yr):________
3. Level of education: diploma B.Pharm MSc
4. Experience in community pharmacy (yr):____________
5. Employment status: employee owner
6. Additional work experience? yes no
7. Are you willing to provide health promotion service to clients? yes no
8. Do you think your professional curricular training is adequate for offering health promotion service?

yes no

**Part II: Involvement in health promotion**

1. **Type of health promotion activity and level of involvement**

| **Activities** | **Level of involvement** | | | |
| --- | --- | --- | --- | --- |
|  | **Very uninvolved** | **Uninvolved** | **Involved** | **Very involved** |
| 1. Asthma counseling |  |  |  |  |
| 1. Diabetes counseling |  |  |  |  |
| 1. Cardiovascular counseling |  |  |  |  |
| 1. Drug misuse |  |  |  |  |
| 1. Nutrition and physical activity |  |  |  |  |
| 1. Smoking cessation counseling |  |  |  |  |
| 1. Oral health |  |  |  |  |
| 1. Immunization |  |  |  |  |
| 1. Traditional medicine counseling |  |  |  |  |
| 1. Weight management counseling |  |  |  |  |
| 1. Family planning |  |  |  |  |
| 1. Cancer counseling |  |  |  |  |

1. How do you evaluate the quality of health promotion service you provided?

poor fair good very good

1. How do you rate your satisfaction with the health promotion service you provided?

very unsatisfied unsatisfied satisfied very satisfied

**Part III: Barriers**

1. **Barriers that limit involvement in health promotion services**

| **Barrier** | **Response** | | | |
| --- | --- | --- | --- | --- |
| 1. Lack of reimbursement from employer or consumers | **SD** | **D** | **A** | **SA** |
| 1. Lack of profitability |  |  |  |  |
| 1. Lack of time |  |  |  |  |
| 1. Lack of training |  |  |  |  |
| 1. Insufficient management support |  |  |  |  |
| 1. Absence of standard guideline for the service |  |  |  |  |
| **SD=strongly disagree; D=disagree; A=agree; SA=strongly agree** | | | | |
